# A Case of Native Mitral Valve Infective Endocarditis in a Chronic Dialysis Patient: The Importance of Vigilance

**DOI:** 10.7759/cureus.69924

**Published:** 2024-09-22

**Authors:** Nyan Myint

**Affiliations:** 1 Internal Medicine, Meharry Medical College, Nashville, USA

**Keywords:** 18fdg pet, 2023 modified duke criteria, acute hypoxic respiratory failure, ct cardiac, left-sided infective endocarditis, mssa endocarditis, persistent mssa bacteremia, transesophageal echocardiography in infective endocarditis, tunnelled dialysis catheter infection

## Abstract

This case involves native mitral valve infective endocarditis in a chronic hemodialysis patient resulting from a catheter-related bloodstream infection (CRBSI). Infective endocarditis in dialysis patients is more common and is associated with higher mortality rates compared to non-dialysis patients. It is also a serious complication of tunneled dialysis catheter infections. The presentation and diagnosis of infective endocarditis in chronic dialysis patients can be challenging and may often be overlooked. This case highlights the importance of maintaining a strong clinical suspicion for infective endocarditis when such patients deteriorate from their baseline status or deviate from the expected response to appropriate treatment.

## Introduction

Infective endocarditis in dialysis patients is 18 times more common and is associated with twice the in-hospital and six-month mortality rates compared to the general population. This increased risk is due to chronic vascular catheters or grafts, repeated vascular access for dialysis, immune dysfunction due to uremia, and associated conditions such as diabetes and valve dysfunction [1]. The leading causative organisms in this population are *Staphylococcus aureus* and *Enterococcus*, with tunneled dialysis catheters or arteriovenous fistulae serving as the major sources of infection [2]. Catheter-related bloodstream infections (CRBSIs), tunnel infections, and exit-site infections are common complications associated with dialysis catheter use. CRBSI is linked to metastatic complications such as infective endocarditis, septic pulmonary emboli, osteomyelitis, and septic arthritis [3]. Diagnosing infective endocarditis can be challenging due to its highly variable multisystemic presentation and the variable knowledge of the condition among physicians. The 2023 Duke ISCVID criteria are typically used to diagnose definitive or possible infective endocarditis [4]. Confirming the presence of vegetation by echocardiography is necessary for diagnosis; both transthoracic echocardiography (TTE) and transesophageal echocardiography (TEE) are widely used. TTE should be performed in all patients with suspected infective endocarditis, but a subsequent TEE should be conducted if TTE results are indeterminate or negative in patients highly suspected of having infective endocarditis. TTE has a lower sensitivity of 70% for native valve endocarditis and 50% for prosthetic valve endocarditis, while TEE has sensitivities of 96% and 92%, respectively [5]. Newer imaging modalities, such as cardiac CT and 18F-fluorodeoxyglucose positron emission tomography-computed tomography (18F-FDG PET/CT), are included in the 2023 modified Duke criteria. This is a case of native valve infective endocarditis in a chronic hemodialysis patient due to a methicillin-sensitive *Staphylococcus aureus *(MSSA) CRBSI.

## Case presentation

A female in her 40s with end-stage renal disease on chronic hemodialysis with chronic tunneled dialysis catheter (at least more than 19 months) presented with acute hypoxic respiratory failure, shortness of breath on exertion, cough with minimal, clear sputum, and generalized body aches without associated fever or chills. The patient received regular dialysis until then without any issues or missed sessions. Physical examination showed bibasilar lung crackles, 1+ bilateral pitting edema with normal heart sounds, no murmurs or added heart sounds, and no jugular venous distension without peripheral signs of IE. Dialysis catheter exit site showed no tenderness or erythema. Blood pressure was 134/70mmHg, heart rate was 85 beats per minute, respiratory rate was 15 breaths per minute, temperature was 99°C, and oxygen saturation was 86% in room air.

Admission labs showed white cell count 12.8x10^9^/L (neutrophils 11.46x10^9^/L, 89.8%), hemoglobin 11.9 g/dL, hematocrit 34.9, platelet count 110x10^9^/L, sodium 123 mmol/L, potassium 5.3 mmol/L, chloride 84 mmol/L, bicarbonate 29 mmol/L, glucose 271 mg/dL, urea 47 mg/dL, creatinine 6.58 mg/dL, eGFR 7 mL/min/1.73m², albumin 3.2 g/dL, calcium 9.9 mg/dL, liver functions normal, lactic acid 1.8 mmol/L, brain natriuretic peptide 22,803 pg/mL, respiratory viral panel negative, and flu and COVID negative. Table 1 shows the laboratory results with reference ranges.

**Table 1:** Admission laboratory results with reference ranges BNP, brain natriuretic peptide; GFR, glomerular filtration rate

Parameter	Value	Units	Reference range
White cell count	12.8	x10^9^/L	4.0-11.0
Neutrophils	11.46	x10^9^/L	2.0-7.0
Neutrophil percentage	89.8%	%	50-70%
Hemoglobin	11.9	g/dL	13.8-17.2
Hematocrit	34.9	%	40-50%
Platelets	110	x10^9^/L	150-400
Sodium	123	mmol/L	135-145
Potassium	5.3	mmol/L	3.5-5.0
Chloride	84	mmol/L	98-106
Bicarbonate	29	mmol/L	22-29
Glucose	271	mg/dL	70-100
Urea	47	mg/dL	7-20
Creatinine	6.58	mg/dL	0.6-1.2
eGFR	7	mL/min/1.73m²	>60
Albumin	3.2	g/dL	3.5-5.0
Calcium	9.9	mg/dL	8.5-10.5
Lactic acid	1.8	mmol/L	0.5-2.2
BNP	22803	pg/mL	<100
Respiratory viral panel	Negative	-	-
Flu A and B	Negative	-	-
COVID-19	Negative	-	-

**Figure 1:**
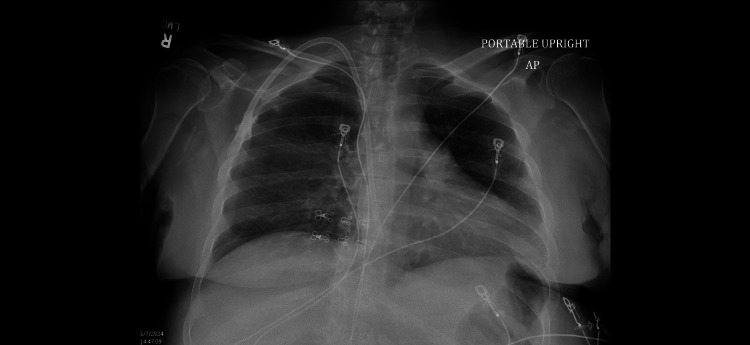
Chest X-ray on admission showing cardiomegaly with left pleural effusion without lung consolidation

Chest X-ray (Figure 1) showed cardiomegaly with left pleural effusion without lung consolidation.

**Figure 2:**
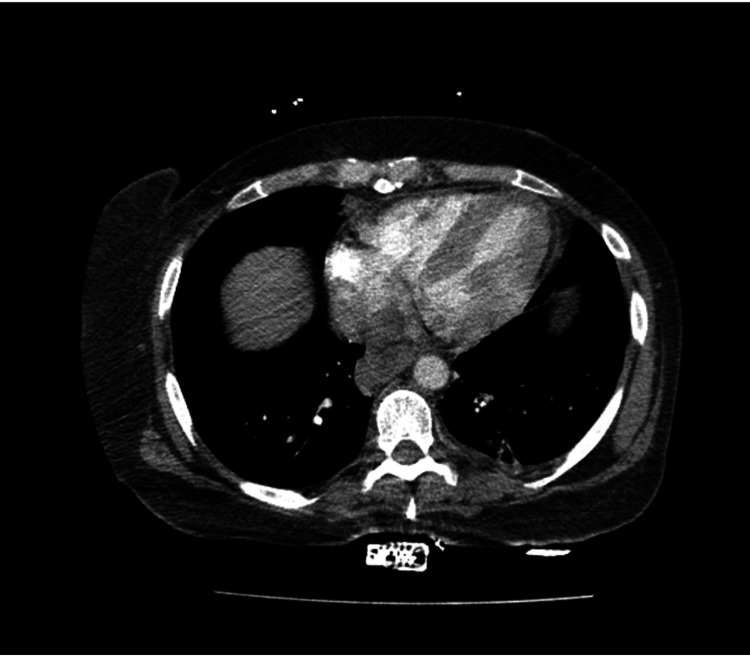
CTA of the chest showing left basilar lung infiltrate with associated minimal left pleural effusion CTA, computed tomography angiography

However, the CT angiogram of the chest showed left basilar lung infiltrate with small left pleural effusion without pulmonary embolus (Figure 2).

IV vancomycin and cefepime were started for broad-spectrum coverage for possible severe community-acquired pneumonia with acute hypoxic respiratory failure. Blood cultures and sputum culture collected on the day of admission were negative. 

**Figure 3:**
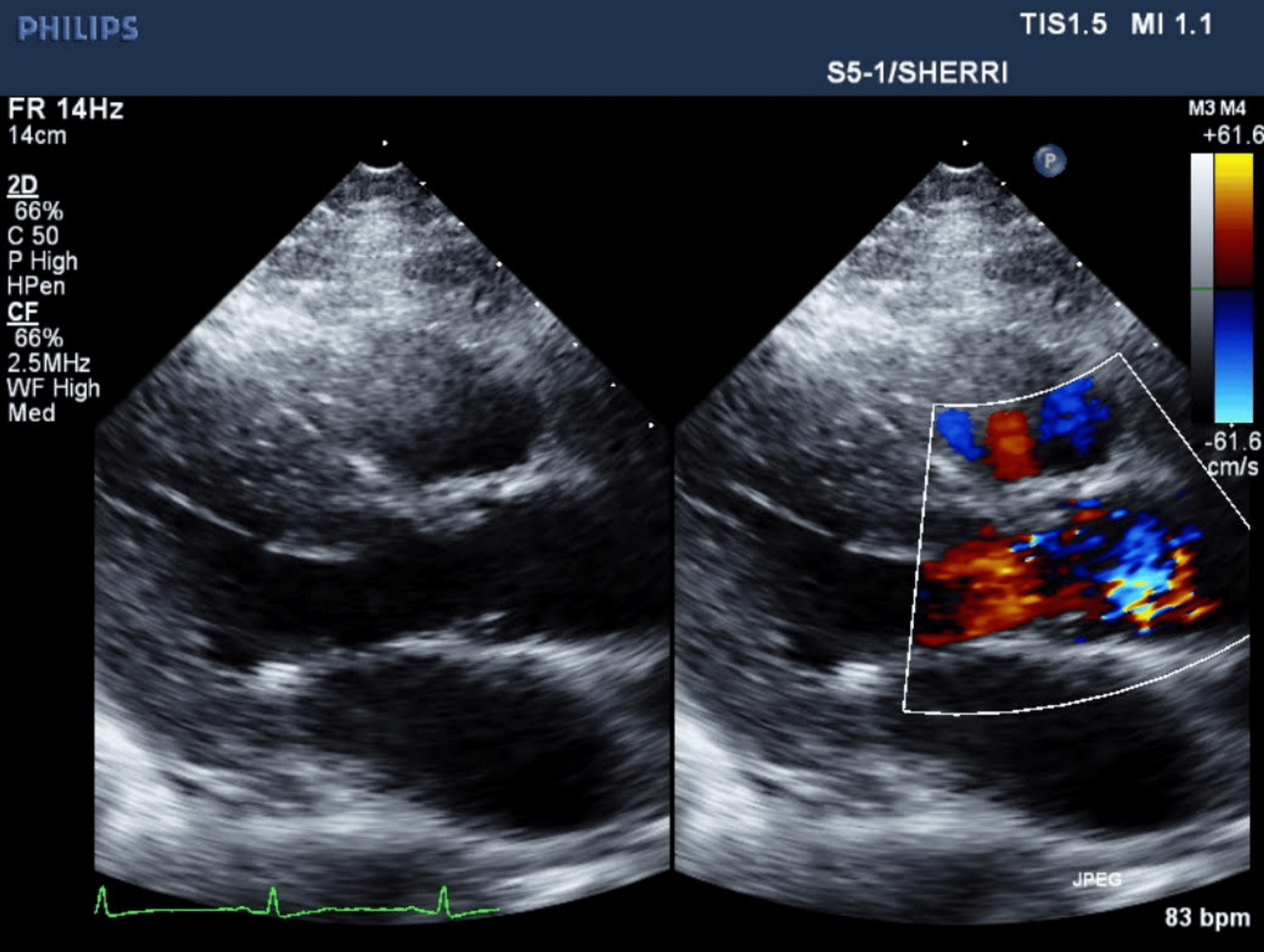
First transthoracic echocardiography since admission with mitral valve visualization without any vegetation

**Figure 4:**
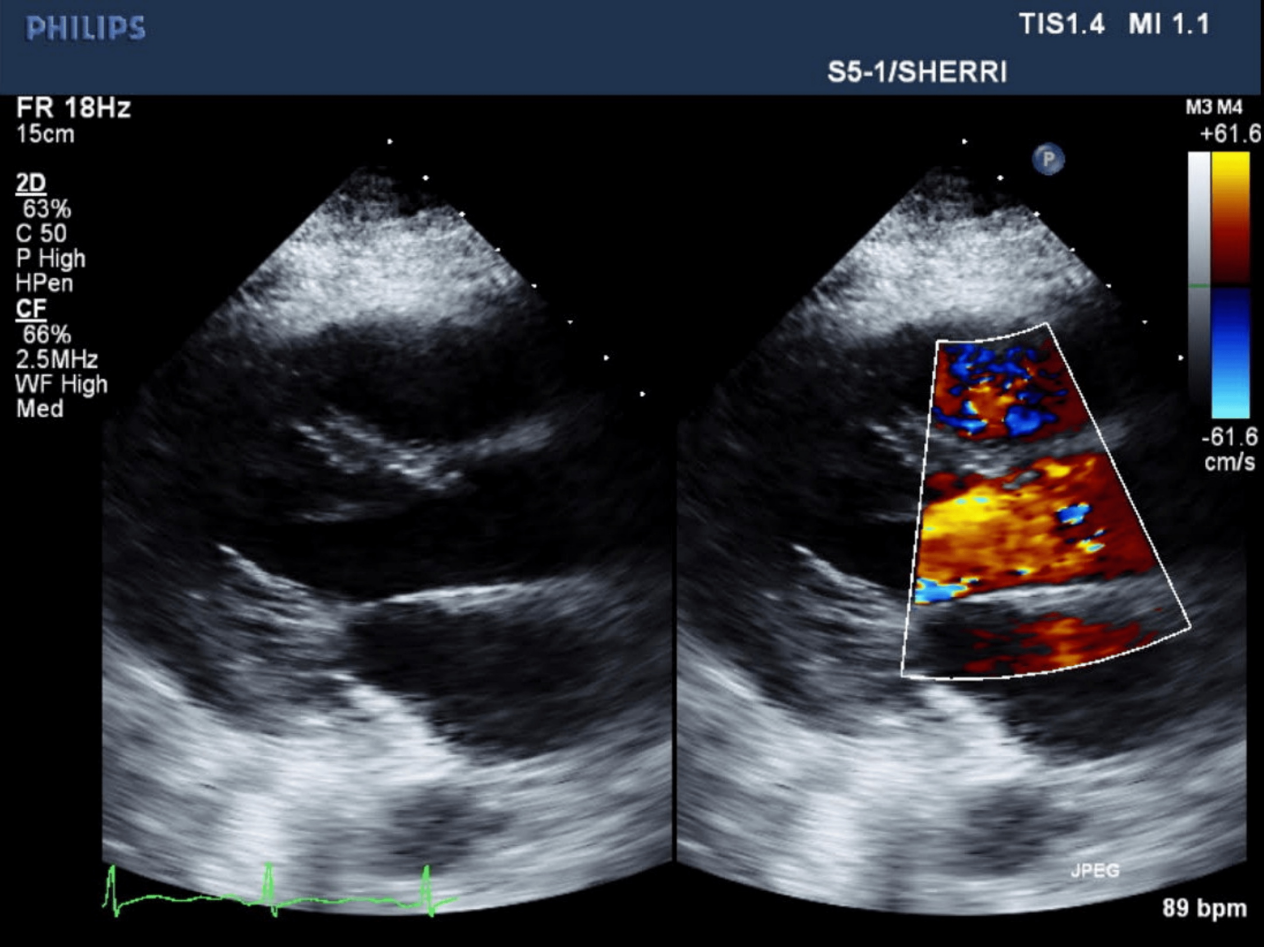
Second transthoracic echocardiography since admission with mitral valve visualization without any vegetation

The patient continued to receive her regular dialysis while inpatient as per her regular schedule. After day 3, the patient's condition declined while on IV vancomycin and cefepime, evident with increase in supplemental oxygen requirement, intolerance to regular dialysis, fever spikes, and worsening leukocytosis. The dialysis tunneled catheter was removed due to risks of CRBSI. MSSA was cultured on the catheter tip and in the blood cultures as well. The infectious disease team was involved in the care at that time. Persistent MSSA bacteremia was diagnosed as repeat blood cultures every other day were positive with the same organisms consistently. First negative culture sets were on day 13 of the hospital course. Initial TTEs performed two times on day 3 since admission (Figure 3) and day 14 since admission (Figure 4) did not show any signs of vegetation or valve regurgitation.

**Figure 5:**
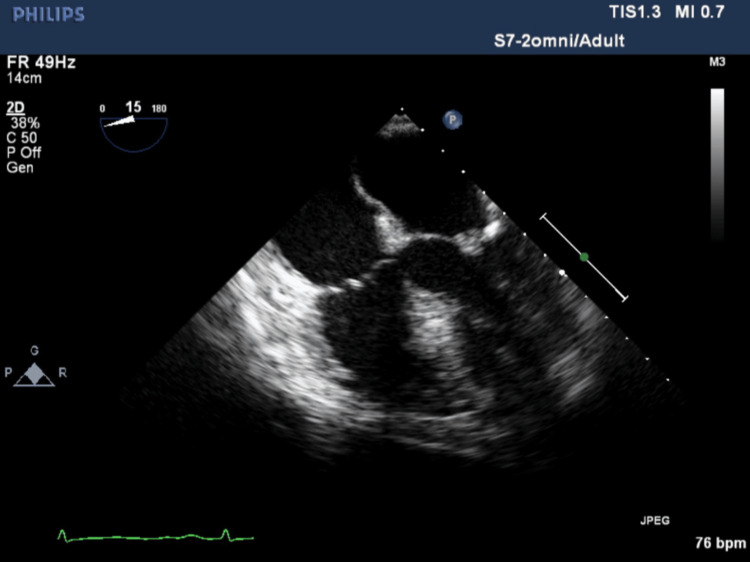
Mitral valve vegetation as small mobile mass on the thickened mitral valve in on transesophageal echocardiogram

Infective endocarditis (IE) was confirmed by the detection of approximately 6mm mobile, vegetation on the mitral valve in TEE on day 19 (seven days after the first negative culture) (Figure 5).

**Figure 6:**
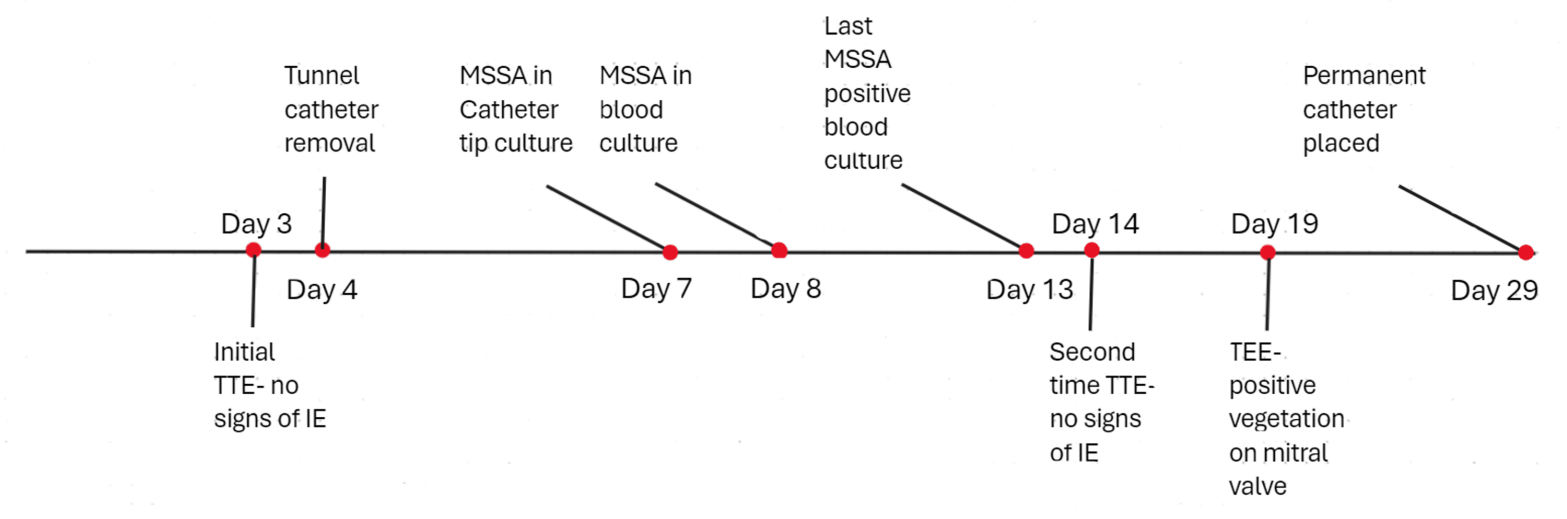
Case timeline MSSA, methicillin-sensitive *Staphylococcus aureus*; TTE, transthoracic echocardiography; TEE, transesophageal echocardiography

The delay for the TEE was due to the patient's unstable vital signs and inability to tolerate the procedure. After 14 days while inpatient, IV vancomycin and cefepime were switched to IV nafcillin according to culture sensitivity. The patient did not develop any complications from IE while inpatient. The patient received a permanent tunneled catheter for dialysis access before discharge. The patient was discharged with a six-week course of IV cefazolin, in accordance with the blood culture sensitivities and the recommendations of the infectious disease team. Figure 6 shows the case timeline.

## Discussion

Diagnosis of definitive or possible IE can be made using the Modified 2023 Duke ISCVD criteria. The criteria consist of major and minor categories, with definitive endocarditis diagnosed by meeting either 2 major criteria, 1 major and 3 minor criteria, or 5 minor criteria. Possible endocarditis is diagnosed by meeting either 1 major and 1 minor criterion or 3 minor criteria [4]. Determining when to apply these criteria can be challenging due to the variable clinical presentation of IE. Therefore, a high suspicion of IE should be considered in all patients with sepsis or fever of unknown origin who have risk factors. IE can present as acute, subacute, or chronic, with or without fever. This non-specific presentation can complicate the diagnostic process. According to the EURO-ENDO registry, the most frequent clinical presentations were fever (77.7%), cardiac murmur (64.5%), and congestive heart failure (27.2%). Classic peripheral signs were less frequently observed but were more common in severe cases of endocarditis. However, vascular and immunological phenomena such as splinter hemorrhages, Roth spots, and glomerulonephritis remained common. Elderly and immunocompromised patients often presented with atypical symptoms [6].

A high suspicion of IE should always be maintained for patients with fever and positive blood cultures without an alternative focus of infection, especially those with one or more risk factors for the disease [7]. Cardiac risk factors include a history of previous IE (highest risk), prosthetic or repaired valves (bioprosthesis>mechanical, especially in mitral and aortic valve), congenital heart disease and first six months after repair procedure for congenital heart disease, cardiovascular implantable electronic device (CIEDs) and left ventricular assist device, and rheumatic, degenerative, and congenital valve disease. Non-cardiac risk factors include central lines, hemodialysis (both tunneled catheter and arteriovenous fistula), recent hospitalization, intravenous drug users, and immunosuppression. Table 2 is a summarization of these risk factors.

**Table 2:** Risk factors for infective endocarditis Data reference: [7] CIED, cardiac implantable electronic device; LVAD, left ventricular assist device

Cardiac Risk Factors	Non-Cardiac Risk Factors
Previous infective endocarditis (highest risk)	Central venous catheter
Valvular heart disease	Intravenous drug users
Prosthetic or repaired valves (bioprosthesis > mechanical)	Immunosuppression
Central arterial or venous catheter	Recent dental or surgical procedures
CIED and LVAD	Recent hospitalization
Congenital heart disease and first six months after repair	Hemodialysis

Staphylococcus aureus is the most common organism causing IE in the United States, accounting for approximately 30% of cases. The incidence of coagulase-negative *Staphylococcus* is also rising in both native and prosthetic valve endocarditis. Oral *Streptococci* (especially *Streptococcus viridans*) account for around 20% of cases, while other *Streptococci* make up approximately 10% of cases. *Enterococci* are responsible for another 10% of cases. HACEK organisms (including *Haemophilus* species, *Aggregatibacter *species, *Cardiobacterium hominis*, *Eikenella corrodens*, and *Kingella* species), fungi, and zoonotic infections together account for less than 5% of cases [8]. Blood culture-negative IE may account for approximately 30% of cases. In two-thirds of these, the causative organisms can be identified through a rigorous diagnostic approach. Serological testing for zoonotic agents such as *Bartonella* species, *Brucella* species, *Coxiella burnetii* (Q fever), *Mycoplasma* species, and *Legionella* species is the initial step. If serological tests are positive, blood polymerase chain reaction (PCR) targeting the causative bacteria should be performed [9]. Table 3 is a summarization of causal organisms for IE and the percentages of associated cases.

**Table 3:** Causal organisms and percentages of associated cases Data reference: [8] HACEK, organisms (*Haemophilus *species, *Aggregatibacter* species, *Cardiobacterium hominis*, *Eikenella corrodens*, and *Kingella* species); IE, infective endocarditis

Causal Organisms	Percentage of Cases
Staphylococcus aureus	30%
Coagulase-negative *Staphylococcus*	Rising (not specified)
Oral *Streptococci *(e.g., *Streptococcus viridans*)	20%
Other *Streptococci*	-
Enterococci	10%
HACEK organisms	<5%
Culture-negative IE	30%

For major microbiological criteria, typical pathogens include positive cultures from two or more blood sets with microorganisms commonly associated with IE, such as *Staphylococcus aureus*, *Staphylococcus lugdunensis*, *Enterococcus faecalis*, all streptococcal species (except *Streptococcus pneumoniae* and *Streptococcus pyogenes*), *Granulicatella* and *Abiotrophia* species, *Gemella* species, and HACEK group microorganisms. In the context of intracardiac prosthetic material, additional bacteria considered as "typical" pathogens include coagulase-negative *Staphylococci*, *Corynebacterium striatum* and *Corynebacterium jeikeium*, *Serratia marcescens*, *Pseudomonas aeruginosa*, *Cutibacterium acnes*, nontuberculous mycobacteria (especially *Mycobacterium chimaera*), and *Candida* species. Non-typical major criteria involve microorganisms that occasionally or rarely cause IE and are isolated from three or more separate blood culture sets [4].

**Figure 7:**
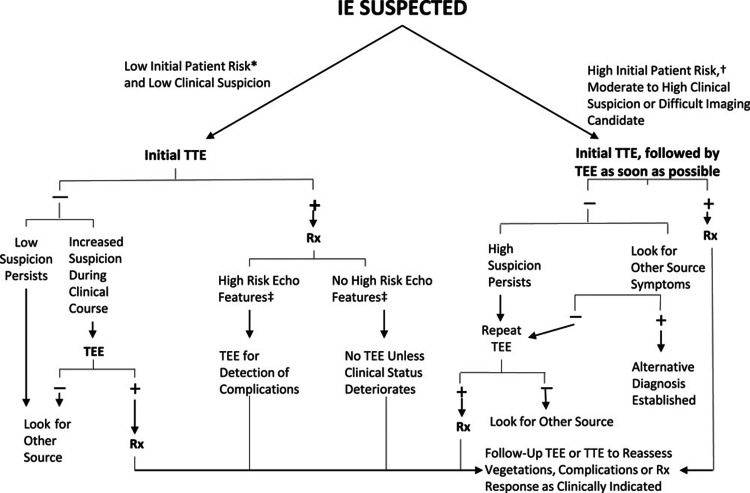
An approach to the diagnostic use of echocardiography *For example, a patient with fever and a previously known heart murmur and no other stigmata of infective endocarditis. †High initial patient risks include prosthetic heart valves, many congenital heart diseases, previous endocarditis, new murmur, heart failure, or other stigmata of endocarditis. ‡High-risk echocardiographic features include large or mobile vegetations, valvular insufficiency, suggestion of perivalvular extension, or secondary ventricular dysfunction. Reproduced from the American Heart Association with permission from original publishers. TEE, transesophageal echocardiography; TTE, transthoracic echocardiography; Rx, treatment

For major imaging criteria, a TTE should be performed in all cases of suspected IE. While TTE has a lower sensitivity (70% for native valves and 50% for prosthetic valves), TEE offers higher sensitivity (96% for native valves and 92% for prosthetic valves) [5]. TEE is also better at detecting smaller vegetations (<1 cm) [10]. If the initial TTE is negative but there remains concern for IE, a TEE should be conducted as soon as possible. Even if the initial TTE is positive, a follow-up TEE is recommended if there are concerns about high risk for intracardiac complications, such as perivalvular extension. Repeating the TEE within three to five days (or sooner if clinical findings change) after an initial negative result is advisable when clinical suspicion of IE persists [11]. Simplification of the approach to the diagnostic use of echocardiography is shown in Figure 7.

Two recently updated imaging modalities in the 2023 modified Duke criteria are cardiac CT scan and 18F-FDG PET/CT. Cardiac CT shows comparable diagnostic performance to TEE for detecting large vegetations (>10 mm) and is more useful for identifying perivalvular abscesses and coronary artery disease. However, TEE has a higher overall detection rate for vegetations (97.3%) compared to CT (72.0%). Additionally, cardiac CT tends to underdiagnose small vegetations (<10 mm) more frequently (52.8%) compared to TEE (94.4%) [12]. Meanwhile, 18F-FDG PET/CT has high specificity for all subtypes of IE but shows lower sensitivity for native valve IE compared to prosthetic valve IE and CIED IE. It can be particularly useful as an adjunct imaging modality for challenging cases of prosthetic valve IE and CIED IE [13].

This case highlights the importance of a meticulous approach in diagnosing IE in patients with predisposing factors. Although the initial presentation and TTEs did not indicate possible IE, a more sensitive TEE was necessary to detect the disease by visualizing small vegetations. Given the high mortality rate, potential complications, and the need for prolonged treatment associated with IE, it is crucial for the care team to maintain a high suspicion for IE in patients presenting with fever and positive blood cultures without an alternative focus of infection, especially if they have one or more risk factors for the disease.

## Conclusions

Variable clinical presentations in patients with IE can derail the physician from making an accurate diagnosis, making it essential for the medical team to maintain a high level of suspicion and use a meticulous approach. Given the high mortality rates, potential complications, and the need for prolonged treatment associated with IE, vigilance is crucial. Understanding the risk factors for IE aids in diagnosis and helps determine whether more invasive imaging, such as TEE, is necessary. The 2023 modified Duke criteria now include newer, less invasive imaging modalities such as cardiac CT scans and 18F-FDG PET/CT. Cardiac CT provides diagnostic performance comparable to TEE for large vegetations (>10 mm) and is particularly useful for detecting perivalvular abscesses and coronary artery disease. However, cardiac CT tends to underdiagnose small vegetations (<10 mm) compared to TEE. Meanwhile, 18F-FDG PET/CT serves as an adjunctive imaging tool for challenging cases of prosthetic valve IE and CIED IE.
